# A Potential Predictor of Proximal Flow-Diverter Stent Jailed Artery Stenosis: Distal to Proximal Artery Diameter Ratio

**DOI:** 10.7150/ijms.115452

**Published:** 2025-06-20

**Authors:** Yudi Tang, Peike Chen, Jian Lv, Haining Wei, Xiaoyan Wang, Junqiang Feng, Yuhua Jiang, Peng Liu, Youxiang Li, Yunna Yang

**Affiliations:** 1 Department of Interventional Neuroradiology, Beijing Neurosurgical Institute, Capital Medical University, Beijing, China.; 2 Department of Neurosurgery, Beijing Tiantan Hospital, Capital Medical University, Beijing, China.; 3 Center for Biomedical Imaging Research, Department of Biomedical Engineering, Medical School, Tsinghua University, Beijing, China.; 4 Department of Biological Sciences, University of California, San Diego, California, United States.; 5 Department of Neurosurgery, Beijing Chaoyang Hospital, Capital Medical University, Beijing, China.

**Keywords:** flow-diverter stent, stenosis, hemodynamics, intracranial artery

## Abstract

Background: Flow-diverting stents (FDS) are widely used in the treatment of intracranial aneurysms. During treatment, many side branches—especially small ones—are inevitably covered, leading to narrowing and impaired blood flow. However, the factors contributing to stenosis have not been thoroughly studied.

Methods: A retrospective cross-sectional study was conducted. Digital subtraction angiography (DSA) images and clinical data were collected. Two neuroradiologists independently assessed arterial geometry characteristics. Participants were divided into a narrow group and unchanged group based on preoperative and follow-up data, and the characteristics of the two groups were compared. A simplified model was established to evaluate changes in wall shear stress (WSS) before and after FDS implantation.

Results: The distal small branch diameter in the unchanged group was significantly smaller than that in the narrow group. The ratio of distal to proximal diameters of the ophthalmic artery (DPOA) in the narrow group was concentrated between 0.9 and 1.1. WSS in the proximal ophthalmic artery region (POAR) increased with larger distal ophthalmic artery diameters, both pre- and post-operatively. The difference between preoperative and postoperative WSS also increased with higher DPOA.

**Conclusions:** After FDS implantation, the WSS of most jailed ophthalmic arteries in POAR decreased. However, in cases with proximal stenosis, the DPOA of these jailed ophthalmic arteries was primarily concentrated between 0.9 and 1.1. This pattern may occur because WSS decreased from high level to low level.

## Introduction

Flow-diverting stents (FDS) are widely used in the treatment of intracranial aneurysms (IAs). However, many side branches are inevitably covered by the stent, a phenomenon referred to as "jailed branches"^1^. While some jailed branches remain unchanged, others may develop stenosis or occlusion^1^. Arterial stenosis or occlusion can potentially lead to ischemic lesions or even permanent neurological impairment^1^-^3^. Given the clinical implications, this phenomenon has garnered increasing attention, with the goal of guiding treatment selection and optimizing therapeutic outcomes with FDS.

Narata *et al.*^1^ analyzed 25 aneurysms and found that stenosis is more likely to occur when the diameter ratio of the two branches is less than 0.7, a finding attributed to hemodynamic factors. Hemodynamic analysis revealed that wall shear stress (WSS)—which is associated with arterial diameter—was significantly reduced following FDS placement, particularly when the ratio of the two branch diameters was less than 0.65. Although the study examined several factors, including branch angles, the analysis of branch diameters was not comprehensive, especially regarding small-diameter jailed branches.

You *et al.*^2^ analyzed anterior cerebral circulation aneurysms and found that the mean transit time and full width at half maximum in covered branches were significantly prolonged post-stenting. However, the study did not control for variables, as all jailed branches were analyzed collectively. Additionally, there was no detailed hemodynamic analysis post-stenting, particularly regarding WSS, which plays a critical role in vascular changes^4^-^9^.

In this study, we focused on the jailed ophthalmic artery (OA) to compare factors between stenotic and non-stenotic jailed branches. Furthermore, we employed a simplified model to analyze hemodynamic changes following stent implantation and to investigate potential mechanisms of stenosis. The findings aim to contribute to the development of more effective treatment strategies in the future.

## Methods

### Study population

The study protocol was reviewed and approved by the local Ethics Committee (Beijing Tiantan Hospital, Capital Medical University). All methods were carried out in accordance with relevant guidelines and regulations in the study and all data were obtained with the informed consent from all subjects and/or their legal guardian(s) in this study.

We retrospectively analyzed participants who underwent flow-diverting stent (FDS) placement with jailed ophthalmic arteries at our center between 2016 and 2020. The Institutional Review Board (IRB) approved the study and waived the further requirement for informed consent due to its retrospective nature. The inclusion criteria were as follows: (1) FDS placement confirmed by digital subtraction angiography (DSA); (2) no prior endovascular treatment before FDS implantation; (3) coverage of OA by the FDS; (4) availability of at least one follow-up DSA; (5) high-quality DSA images with clear visualization of the OA and internal carotid artery (ICA). and (6) the participant or their guardian signed the informed consent form, agreeing that their clinical data, after removing identifiable information, could be used for future research. Participants who underwent more than one FDS implantation or with mismatch stent were excluded from the study. Demographic characteristics, aneurysm features, and treatment-related data were systematically collected and analyzed.

### Treatment management

Participants received dual antiplatelet therapy (aspirin 100 mg/day and clopidogrel 75 mg/day) at least five days before the procedure. Preoperative platelet function tests were performed, and clopidogrel non-responders were switched to ticagrelor (90 mg twice daily). All FDS implantations were performed via the femoral artery under general anesthesia. The treatment strategy (FDS alone or FDS with coils) was determined based on aneurysm anatomy and operator experience. Post-procedure, dual antiplatelet therapy was maintained for at least six months, followed by single antiplatelet therapy (aspirin, clopidogrel, or ticagrelor) for at least one year. Medication plans were personalized by the attending physician.

### DSA acquisition

All DSA acquisitions follow standardized angiography and contrast agent injection protocols, which are consistently applied to all participants. A 6F catheter is positioned at the origin of the internal carotid artery, and an iodine-based contrast medium is administered via power injection at a rate of 4 ml/s, resulting in a total volume of 6 ml. DSA images are acquired at a frame rate of 4 frames per second throughout the procedure.

### Data measurement

Two neuroradiologists (13 and 14 years of experience) independently measured artery diameters on pre-stent and follow-up images. The final diameter was the average of their measurements. Discrepancies were resolved by a third neuroradiologist (22 years of experiences based on the imaging findings, medical records, and clinical experience.

### Parameter measurement

Diameters were measured at the following locations using pre-stenting images: proximal trunk diameter (proximal diameter of the horizontal section of the cavernous segment of ICA), distal trunk diameter (at the junction of the clinoid segment of ICA and the OA), proximal main branch diameter (at the junction of the ophthalmic segment of ICA and the OA), distal main branch diameter (at the junction of the ophthalmic segment of ICA and the posterior communicating artery), proximal small branch diameter (at the beginning of the long limb of the OA), and distal small branch diameter (at the beginning of the second part of the intraorbital segment of the OA). The segments of ICA were divided according to the Bouthillier segmentation method^10^. And the segments of OA were divided according to the Hayreh's method^11^-^13^.

### Grouping

By comparing the DSA images before FDS implantation and at the last follow-up, participants with obvious stenosis in the proximal ophthalmic artery region (POAR) were divided into the narrow group, and other participants were divided into the unchanged group. Participants were further categorized into a stent stenosis group and a non-stent stenosis group. Stent stenosis was defined as a reduction in arterial diameter of more than 50%.

In addition, participants were also divided into the progressive symptom group and the non-progressive symptom group. Progressive symptoms were defined as clinical symptoms that were inconsistent with or more severe than those before stent implantation, with no other identifiable causes found through clinical evaluation.

### Simplified model

**Figure 1:**
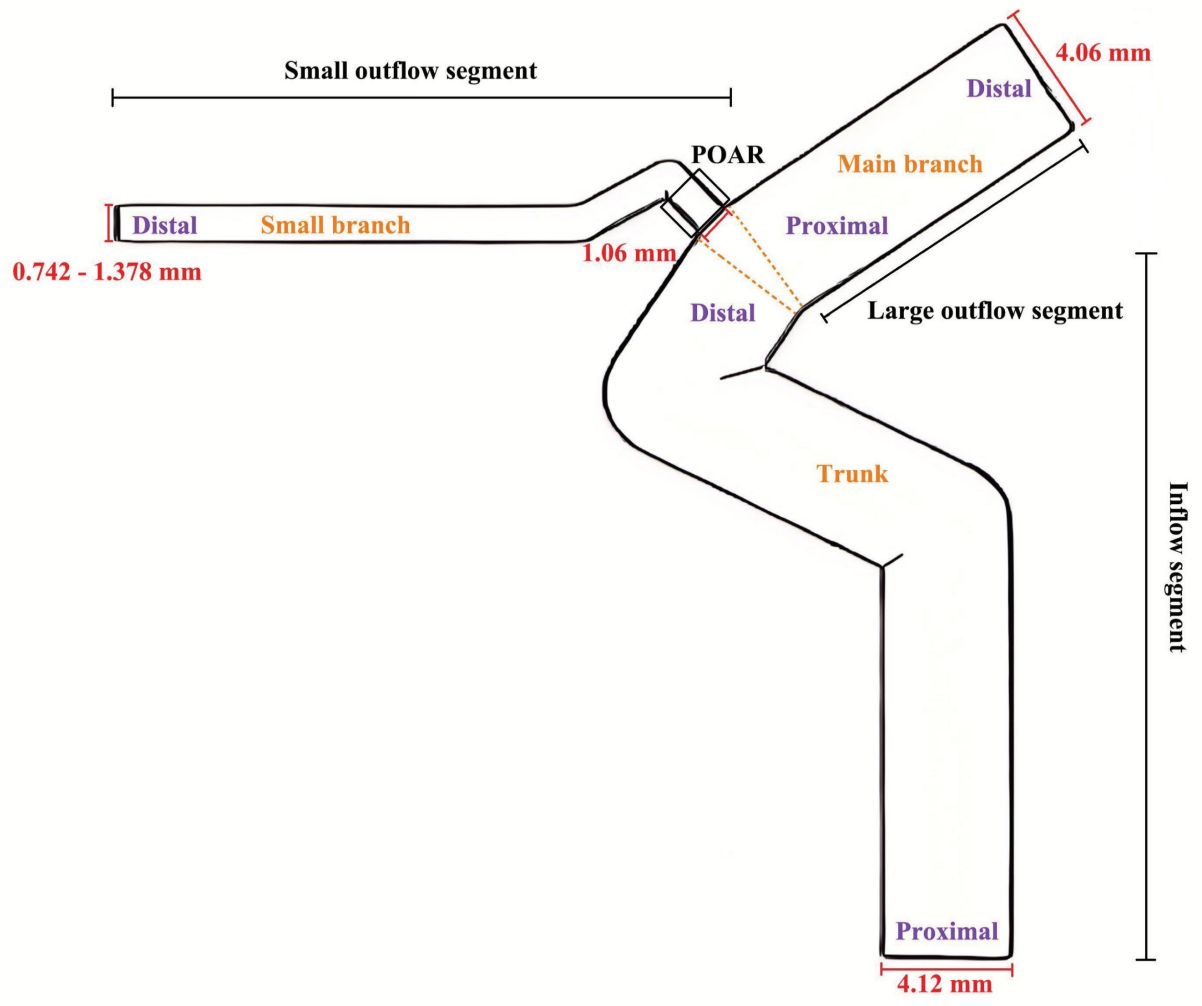
Schematic diagram of simplified models of the ophthalmic artery and internal carotid artery,

Based on previous studies^11^-^15^ and the results of this study, simple three-dimensional models were established using FreeCAD 0.18. Significant influencing factors on hemodynamics were calculated and analyzed based on the observation results. The diameter of the inflow segment was 4.12 mm, and its length was 30 mm. The diameter of the large outflow segment was 4.09 mm, and its length was 15 mm. The diameter of the proximal small outflow segment was 1.06 mm, while the diameter of the distal small outflow segment ranged from 0.742 to 1.378 mm (DPOA: 0.7 to 1.3). Simplified models were constructed for each 0.053 mm (DPOA: 0.05) change in the diameter of the distal small outflow segment. The length of the small outflow segment was 20 mm. All bending angles in the model were derived from previous studies^12^, ^14^. A diagram is shown in Figure 1. Additionally, an approach previously described by Larrabide *et al.*^16^ was used to build models with FDS implantation.

### Hemodynamics

OpenFOAM-11, using the incompressible transient solver, was used to analyze hemodynamics. Blood was modeled as an incompressible Newtonian fluid with a density of 1054 kg/m³ and a dynamic viscosity of 3.5 mPa·s. Vascular walls were assumed to be rigid and non-slip. A pulsatile physiological flow waveform measured in the ICA from a previous study^17^ was used to impose boundary conditions at the inlet, and pressure boundary conditions reported by Reymond *et al.*^18^ were applied at the outlet. Three cardiac cycles were simulated, and the results of the third cycle were analyzed.

### Statistical analysis

Statistical analyses were conducted using R. The normality of data distribution was tested using the Kolmogorov-Smirnov and Shapiro-Wilk methods. Correlation was examined using Pearson's or Spearman's method according to distribution type. Chi-Square Pearson test, correction for continuity and Fisher's exact test were used to compare morphological features between stenosis and unchanged group. The rank sum test or t test is used to compare the difference between two sets of values. P <0.05 was considered significant.

## Results

This study included a total of 145 participants (30 in the narrow group and 115 in the unchanged group) with an average follow-up period of 12.6 months.

### Participant characteristics

As shown in Table 1, there were no significant differences between the two groups in gender (p=0.305), smoking status (p=0.793), alcohol consumption (p=1.000), hypertension (p=0.493), hyperlipidemia (p=0.681), height (p=0.928), weight (p=0.306), pre-SAH history (p=0.961), pre-stroke history (p=1.000), follow-up duration (p=0.301), or stent stenosis (p=0.345). However, compared with the unchanged group (mild intermittent headache, 4; slightly dizzy, 2), participants in the narrow group (mild intermittent headache, 6; slightly dizzy, 1; decreased vision, 2; feeling uncomfortable but indescribable, 1) had a significantly higher proportion of participants with symptom progression (p<0.001).

### Arterial diameter

There were no significant differences between the narrow group and unchanged group in proximal trunk diameter (p=0.866), distal trunk diameter (p=0.744), proximal main branch diameter (p=0.764), distal main branch diameter (p=0.918), or proximal small branch diameter (p=0.099). The distal small branch diameter in the unchanged group was significantly smaller than that in the narrow group (p=0.033). More details are shown in Table 1.

There were also no significant differences between the progressive symptom group and non-progressive group in proximal trunk diameter (p=0.724), distal trunk diameter (p=0.970), proximal main branch diameter (p=0.960), distal main branch diameter (p=0.714), proximal small branch diameter (p=0.704), and distal small branch diameter (p=0.329). More details are shown in Table 2.

### Arterial diameter ratio

**Figure 2:**
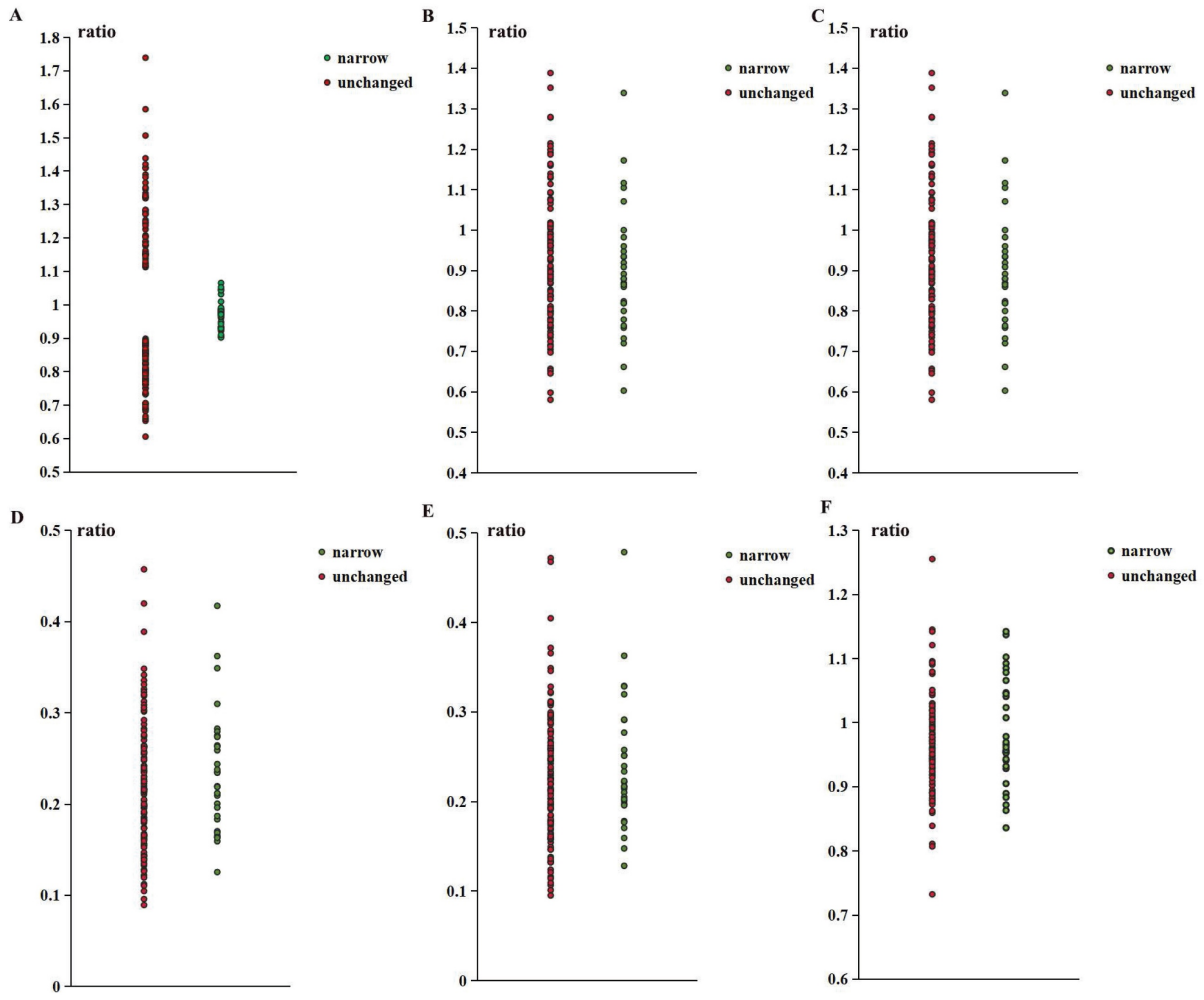
The ratio distribution of narrow group and unchanged group. (A. Distal small branch diameter / proximal small branch diameter; B. Distal main branch diameter / proximal main branch diameter; C. Distal trunk diameter / proximal trunk diameter; D. Proximal small branch diameter / distal trunk diameter; E. Proximal small branch diameter / proximal main branch diameter; F. Proximal main branch diameter / distal trunk diameter).

As shown in Table 1, there were no significant differences between the two groups in any arterial diameter ratios. However, the ratio of distal to proximal ophthalmic artery (DPOA) in the stenosis group was concentrated between 0.9 and 1.1. The association between this range and the stenosis group was significant. More details of the ratio distribution are shown in Figure 2.

### Progressive symptoms

There were 16 participants (mild intermittent headache, 10; slightly dizzy, 3; decreased vision, 2; feeling uncomfortable but indescribable, 1) divided into progressive symptom group. The proportion of participants with POAR stenosis in the progressive symptom group was significantly higher than that in the non-progressive symptom group(p<0.001). There was no statistically significant difference in the proportion distribution of participants with stent stenosis between the two groups (p=1.000). More details were shown in Table 2.

### Hemodynamics

**Figure 3:**
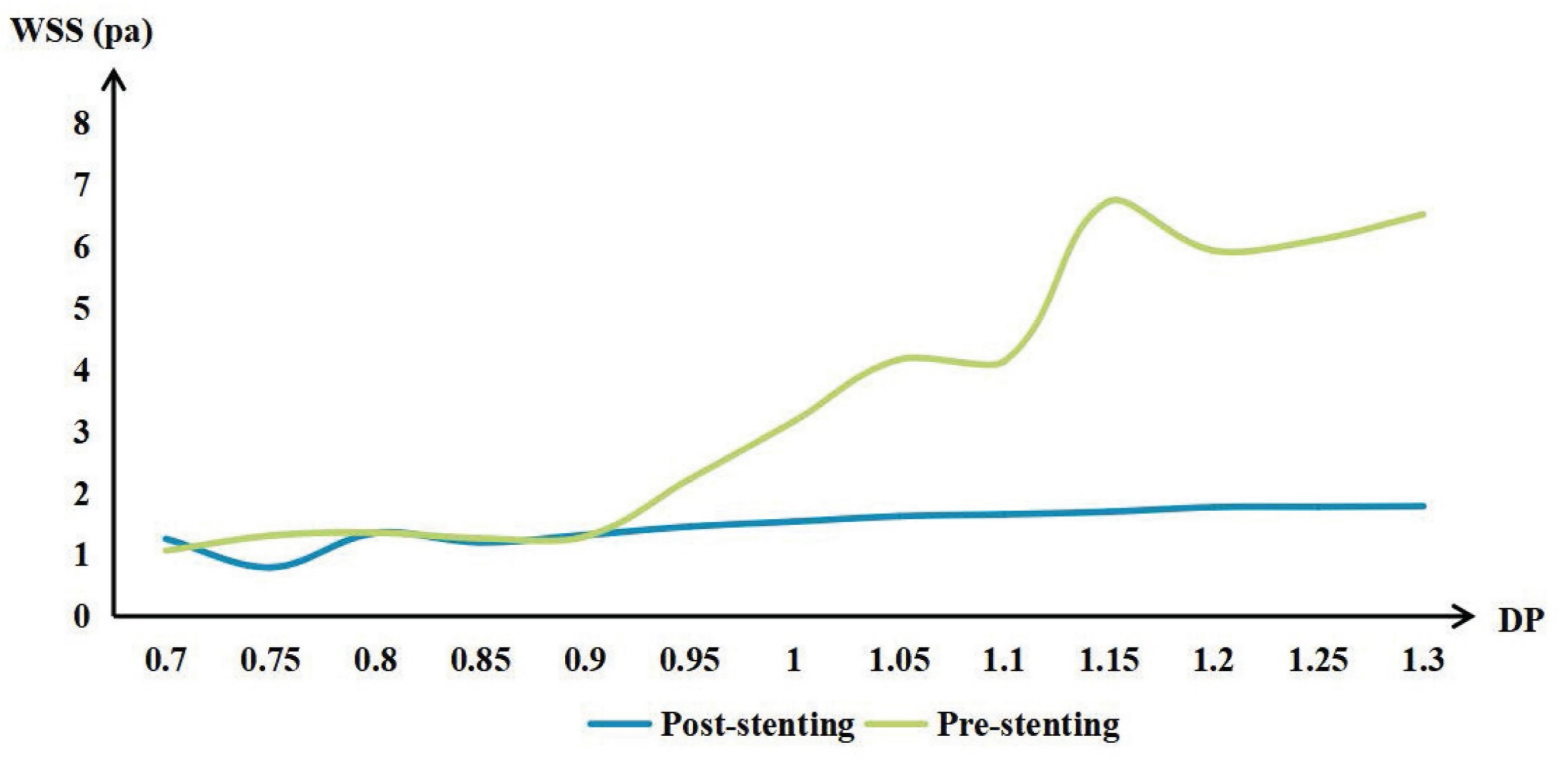
The mean wall shear stress (WSS) of the proximal ophthalmic artery region (POAR) with different distal-to-proximal ophthalmic ratio (DPOA) before and after stenting.

WSS in the POAR increased with increasing distal OA diameter, both preoperatively and postoperatively. The difference of preoperative and postoperative WSS also increased with the increase of DPOA. More details are shown in Figure 3.

## Discussion

DPOA has a significant influence on the lumen diameter changes of POAR during the follow-up period in this study. This phenomenon may be caused by changes in WSS before and after stenting. Different DPOA values can lead to varying WSS changes. WSS is widely recognized to play a critical role in regulating arterial diameter and vascular remodeling^1^, ^4^-^9^.

WSS regulates arterial diameter by affecting endothelial function, primarily through the increased secretion of prostacyclin and nitric oxide^19^. Decreased peripheral vascular resistance and increased blood flow demand lead to increased blood flow velocity and increased WSS. Subsequently, increased WSS triggers the release of nitric oxide, causing vasodilation and restoring baseline WSS levels. If such WSS changes persist, the artery will gradually remodel^20^, ^21^. This regulatory mechanism has been confirmed in in vivo experiments^7^, ^21^, ^22^. Notably, low WSS resulting from FDS implantation can disrupt this process, reducing nitric oxide release and leading to arterial constriction and remodeling. This mechanism may explain the observed decrease in POAR diameter.

Most participants with a DPOA less than 0.9 exhibited minimal changes in WSS after stent implantation in the POAR. These subtle WSS changes resulted in only minor or no arterial diameter changes, as WSS remained at baseline levels. Interestingly, participants with a DPOA of around 0.75 also showed a significant decrease in WSS after FDS implantation, but no stenosis was observed in this group. However, only a small number of participants fall within this range, and this finding requires further investigation. Additionally, participants with smaller DPOA values generally experienced smaller declines in WSS, which may explain why the distal small branch diameter was smaller in the unchanged group.

Although participants with a DPOA greater than 1.1 showed significant decreases in WSS, many were categorized into the unchanged group. Several hypotheses may explain this observation. First, while WSS decreased, it remained at or above baseline levels^23^. These participants typically had higher baseline WSS, and the changes induced by FDS implantation may have prevented or delayed arterial enlargement. Compared to participants with a DPOA smaller than 0.9, those with a DPOA greater than 1.1 had similar or even higher WSS values in this study. Second, chronic high WSS can damage the arterial wall^20^, leading to functional impairment and irreversible dilation. Endothelial cells produce matrix metalloproteinases, which degrade the extracellular matrix, and growth factors that stimulate cell proliferation. As WSS decreases, proliferating cells migrate into newly enlarged areas, allowing smooth muscle cells to reorient and adapt to arterial enlargement^23^. For example, in intracranial aneurysms, high WSS contributes to aneurysm formation and growth, while subsequent low WSS often leads to wall thickening and inflammation rather than recovery^24^, ^25^. Third, higher blood flow volume increases distal peripheral resistance, providing a greater compensatory reserve. Participants with a higher DPOA typically have higher pre-stenting blood flow velocities, and reductions in peripheral vascular resistance are often accompanied by increased blood flow and WSS^21^. However, these hypotheses require further validation through in vivo experiments. Additionally, proximal stenosis may transition to a larger DPOA.

Narata *et al.*^1^ analyzed hemodynamic changes based on branch angles and diameter ratios. They found that diameter ratios were associated with small branch stenosis due to hemodynamic changes, particularly WSS reduction, while no significant correlation was observed with branch angles. However, their study had a small sample size and lacked analysis of minor branches, which are clinically significant. In this study, we observed similar phenomena in jailed OA using a larger sample and explored potential mechanisms. Nevertheless, our findings cannot be generalized to all minor branches, and the exact mechanisms in other arteries require further investigation. The conclusions drawn from this study also need in vivo validation.

You *et al.*^2^ studied hemodynamic changes using quantitative digital subtraction angiography after FDS implantation in the anterior circulation. They found that FDS implantation reduced blood flow in jailed branches, while flow-impaired branches exhibited increased blood flow velocity compared to patent branches. Quantitative digital subtraction angiography provided real blood flow data, moving beyond theoretical analysis. However, their study did not assess WSS, a critical factor in arterial diameter changes, due to methodological limitations. The increased blood flow velocity in flow-impaired branches after FDS implantation may indicate reduced distal peripheral resistance as a compensatory response to WSS reduction. However, WSS changes cannot be inferred solely from blood flow velocity^26^, especially when blood flow is not parallel to or near the arterial wall. In this study, we observed similar reductions in blood flow after FDS implantation and further evaluated WSS changes. However, the model established in this study is simplified without many details, and there might even be many potential influencing factors that have not been recognized. Clearly, the results in this study can only be used as a reference and need to be constantly verified.

Progressive symptom was more likely observed in the narrow group and may be associated with POAR stenosis. Decreased vision may be caused by reduced blood flow in the OA. Headache and dizziness may be caused by insufficient blood supply in some areas when the lateral branches of the eye blood flow compensate. However, it is important to note that stent implantation is the main factor leading to reduced blood flow rather than stenosis. Most progressive symptoms can be caused by a variety of common factors, even if they are not found during follow-up examinations. Furthermore, for participants with the same symptoms before and after FDS implantation, the causes of the symptoms may be different. Clearly, the clinical significance of POAR stenosis needs to be further explored.

Additionally, focusing solely on OA minimized confounding factors such as embryonic development, surrounding anatomy, and supplying arteries, improving the accuracy of group classification. However, the complexity of blood flow environments and variables makes it challenging to generalize to other jailed branches. Compared with the posterior circulation, the results of this study may be of greater reference value for research on incarcerated arteries in the anterior cerebral circulation, as they may have more similar microstructures to autonomic control of cerebral blood flow^27^. During embryonic development, the OA and anterior circulation arteries originate from the third aortic arch and proximal dorsal aorta, while the posterior circulation originates from the longitudinal neural arteries and intersegmental arteries. However, the differentiation, anastomosis, and degeneration of arteries are constantly occurring, and each artery is unique. Clearly, more advanced methods and larger sample sizes are essential for future studies to yield more accurate and generalizable results.

### Limitations

This study has several limitations. First, it is a single-center, retrospective study. The limited population may affect the generalizability of the findings. Second, stent implantation was performed by multiple operators, and the doctors and nurses involved in the treatment were not fixed and could not be standardized. The bias caused by this confounding factor is uncontrollable and cannot be evaluated. Third, hemodynamic data were not obtained from in vivo experiments. Although computational fluid dynamics is widely recognized in the analysis of hemodynamics, there are still differences between the results and reality, especially in simplify models. Finally, only OA was included in this study. Although this approach can reduce the interference of confounding factors and make the results more reliable, it can only serve as a reference when analyzing other branches. A multicenter prospective cohort study with a larger sample size, longer follow-up time, and more robust methods would avoid these limitations in the future.

## Conclusion

OA with a DPOA between 0.9 and 1.1 was more likely to develop proximal artery stenosis after FDS implantation. This phenomenon may be caused by decreased WSS. However, WSS reduction was observed in most jailed OA cases, not only in participants with proximal artery stenosis.

## Figures and Tables

**Table 1:** Comparison of participant characteristics, arterial diameter, arterial diameter ratio between unchanged and narrow group

	Unchanged(n=115)	Narrow(n=30)	p
Participant characteristics			
Female	78.3%	86.7%	0.305
Hypertension	36.5%	43.3%	0.493
Diabetes	9.6%	6.7%	0.621
Hyperlipidemia	37.4%	33.3%	0.681
Pre-SAH	4.3%	6.7%	0.961
Pre-stroke	5.2%	6.7%	1.000
Alcohol	11.3%	10.0%	1.000
Smoke	13.9%	10.0%	0.793
Stent stenosis	5.2%	0.0%	0.345
Height, median (25%,75%) (cm)	162 (158,167)	161 (159,166)	0.928
Weight, median (25%,75%) (kg)	64 (58,70)	65 (59,75)	0.306
Age, median (25%,75%) (years)	53 (46,59)	56 (50,62)	0.159
Follow-up, median (25%,75%) (months)	9 (7,18)	8 (6,14)	0.301
Symptom progression	5.2%	33.3%	<0.001
Arterial diameter			
Proximal trunk diameter, median (25%,75%) (mm)	4.13 (3.63,4.62)	4.19 (3.54,4.68)	0.866
Distal trunk diameter, median (25%,75%) (mm)	4.11 (3.53,4.62)	3.95 (3.48,4.53)	0.744
Proximal main branch diameter, median (25%,75%) (mm)	3.99 (3.19,4.55)	3.95 (3.47,4.54)	0.764
Distal main branch diameter, median (25%,75%) (mm)	3.48 (3.09,4.08)	3.59 (3.20,3.95)	0.918
Proximal small branch diameter, median (25%,75%) (mm)	0.84 (0.66,1.07)	0.93 (0.77,1.16)	0.099
Distal small branch diameter, median (25%,75%) (mm)	0.79 (0.67,0.95)	0.97 (0.72,1.07)	0.033
Arterial diameter ratio			
Distal trunk diameter / proximal trunk diameter, median (25%,75%)	0.952 (0.861,1.087)	0.967 (0.853,1.040)	0.781
Distal main branch diameter / proximal main branch diameter, median (25%,75%)	0.908 (0.805,1.011)	0.886 (0.795,0.985)	0.507
Distal small branch diameter / proximal small branch diameter, median (25%,75%)	0.866 (0.800,1.189)	0.973 (0.931,0.996)	0.060
Proximal small branch diameter / distal trunk diameter, median (25%,75%)	0.219 (0.173,0.276)	0.228 (0.197,0.288)	0.089
Proximal main branch diameter / distal trunk diameter, median (25%,75%)	0.960 (0.920,1.006)	0.968 (0.939,1.052)	0.178
Proximal small branch diameter / proximal main diameter, median (25%,75%)	0.213 (0.165,0.262)	0.236 (0.180.0.274)	0.234

**Table 2:** Comparison of the proportions of proximal arterial stenosis and stent stenosis between the progressive symptom group and the non-progressive symptom group

	Progressive symptom group (n=16)	Non-progressive symptom group (n=129)	p
Proximal ophthalmic artery region stenosis	62.5%	15.5%	<0.001
Stent stenosis	6.3%	3.9%	1.000
Proximal trunk diameter, median (25%,75%) (mm)	4.27 (3.65,4.72)	4.11 (3.62,4.62)	0.724
Distal trunk diameter, median (25%,75%) (mm)	4.23 (3.52,4.66)	3.99 (3.52,4.61)	0.970
Proximal main branch diameter, median (25%,75%) (mm)	4.18 (3.17,4.66)	3.96 (3.25,4.47)	0.960
Distal main branch diameter, median (25%,75%) (mm)	3.62 (3.22,4.09)	3.46 (3.09,4.04)	0.714
Proximal small branch diameter, median (25%,75%) (mm)	0.86 (0.65,1.01)	0.87 (0.66,1.10)	0.704
Distal small branch diameter, median (25%,75%) (mm)	0.93 (0.68,0.99)	0.79 (0.67,0.99)	0.329

## Abbreviations

FDS: flow-diverting stents
DSA: digital subtraction angiography
WSS: wall shear stress
DPOA: The ratio of the distal to proximal diameters of the ophthalmic artery
POAR: proximal ophthalmic artery region
IAs: intracranial aneurysms
OA: ophthalmic artery
